# Quinoa–Peanut Relay Intercropping Promotes Peanut Productivity Through the Temporal Optimization of Soil Physicochemical Properties and Microbial Community Composition in Saline Soil

**DOI:** 10.3390/plants14142102

**Published:** 2025-07-08

**Authors:** Xiaoyan Liang, Rao Fu, Jiajia Li, Yinyu Gu, Kuihua Yi, Meng Li, Chuanjie Chen, Haiyang Zhang, Junlin Li, Lan Ma, Yanjing Song, Xiangyu Wang, Jialei Zhang, Shubo Wan, Hongxia Zhang

**Affiliations:** 1 Shandong Institute of Sericulture, Shandong Academy of Agricultural Sciences, Yantai 265503, China; liangxiaoyan1001@163.com (X.L.); frao2017@163.com (R.F.); jjli7525@163.com (J.L.); guyy70@163.com (Y.G.); kuihuayi1016@163.com (K.Y.); lim0320@126.com (M.L.); chuanjie79@163.com (C.C.); oceanz_zhy@163.com (H.Z.); lijunlin517@163.com (J.L.); cysmalan@shandong.cn (L.M.); yjsong1214@163.com (Y.S.); cyswangxiangyu@shandong.cn (X.W.); 2 National Center of Technology Innovation for Comprehensive Utilization of Saline-Alkali Land, Dongying 257345, China; 3 State Key Laboratory of Nutrient Use and Management, Institute of Agricultural, Resources and Environment, Shandong Academy of Agricultural Sciences, Jinan 250100, China; 4 Shandong Academy of Agricultural Sciences, Jinan 250100, China; zhangjialei19@163.com; 5 The Engineering Research Institute of Agriculture and Forestry, Ludong University, Yantai 264025, China

**Keywords:** quinoa, peanut, relay intercropping, soil property, microbial community

## Abstract

Peanut productivity is severely restricted by soil salinization and associated nutrient deficiency in saline soil. The quinoa–peanut relay intercrop pattern (IP) is a promising planting system that utilizes the biological advantages of quinoa to improve soil ecological functions and productivity. However, the effects of IP on soil physicochemical and biological properties and the yield formation of the combined peanut crop are still unclear. Two-year field experiments in coastal saline soil were conducted to explore the effects of IP on peanut growth and pod yield, soil physicochemical properties, and microbial community characterization at different growth stages of peanut based on the traditional monocrop pattern (MP). The results show that IP promoted peanut pod yield, although there was the disadvantage of plant growth at an early stage. Soil water content, electrical conductivity (EC), and Na^+^ content in the peanut rhizosphere were lower, whereas K^+^, NH_4_^+^, and total organic carbon (TOC) contents were higher in IP systems at both the vegetative and reproductive stages. The pod yield of peanut was significantly negatively correlated with soil EC and Na^+^ contents at the vegetative stage, but positively correlated with K^+^, NO_3_^−^, NH_4_^+^, PO_4_^3−^, and TOC contents at the reproductive stage. IP rebuilt the composition of the soil bacterial community in the peanut rhizosphere and increased the abundance of the beneficial bacterial community, which were positively correlated with soil TOC, K^+^, NH_4_^+^, NO_3_^−^, and PO_4_^3−^ contents. These findings suggest that IP can increase peanut pod yield through optimizing soil physicochemical properties and microbial community composition, and it is a promising planting system for improving agricultural production in coastal saline lands.

## 1. Introduction

Soil salinization has become an ever-increasing threat to global agricultural production and environmental ecology [1,2]. The main reasons leading to low plant productivity in saline lands are the growth inhibition caused by a high sodium (Na^+^) concentration and associated poor soil physicochemical properties [3]. A high concentration of salt in soil affects a series of processes related to soil fertility and soil ecological functions mediated by microorganisms, which further lead to the deterioration of the soil environment [4]. Intercropping with halophytes is a promising method using the “biological salt removal” of halophytes to improve crop yield and ecological function in saline–alkali soil [5]. In order to survive in saline soil, halophytes absorb salt ions from soil solutions to adjust the osmotic potential, thereby reducing Na^+^ accumulation in the soil and alleviating the salt stress of combined crops in intercropping systems [6,7,8]. In addition to soil salinity, the yield of intercrops is also limited by soil nutrients, such as nitrogen, phosphorus, and potassium, and soil water availability [9].

Intercropping is an efficient planting pattern in which two or more crops are planted simultaneously in one field during the same or part of their growing time [10]. Compared with monoculture, intercropping can give full play to the advantages of niche complementarity and the interspecific facilitation of combined species, improve resource utilization efficiency, and thus improve agricultural productivity [2]. When intercropped species are sown and/or harvested at different times, it is called relay intercropping, which is different from intercropping, with completely overlapping growth periods [11]. Due to the weaker competitive strength of the later-sown crop during the overlapping period, the species sown first usually dominates the species sown later in a relay intercropping system [12]. However, after the harvesting of the first-sown crops, the crops sown later usually exhibit a faster growth rate than those in single cropping, because there is more availability of light and belowground resources per plant [13]. The yield of crops sown later depends on the strength of the “competition–recovery effect” after the harvesting of the early-maturing crops [14]. Compared with intercropping, relay intercropping has more advantages in the utilization of resources, because it possesses the advantage of temporal niche differentiation, and both crops share different growth periods with a complementary resource use of nutrients [15]. In the relay intercropping system, the spatial distribution of soil organic matter and total nitrogen can increase, leading to improved soil fertility [16].

The microbial community is thought to be a key component of the rhizosphere soil ecosystem and has been used to evaluate soil quality [17]. In the rhizosphere, 95% of microorganisms belong to bacteria, which are often capable of colonizing the ecological niche on the growing roots of plants [18,19]. The adaptive evolution and composition of a microbial community are regulated by plant species, plant developmental stages, and environmental factors [20]. To obtain carbon and energy from the root exudates of plants, bacteria inhabit the rhizosphere soils of plants, but rhizosphere bacteria have a certain specificity to plants. The complicated plant diversity is beneficial to the construction of a more stable and healthy soil microbial community [21,22]. Compared to monoculture, intercropping is known to alter rhizosphere microbial community characteristics, such as microbial community composition [23,24]. Mulberry–soybean intercropping increased the abundance of beneficial bacteria in saline soils [25]. Similarly, maize–cassava relay intercropping reshaped the beneficial bacterial community related to soil nutrient cycling [26].

Quinoa is a typical halophyte species widely planted in saline–alkali soil due to its unique nutritional value and outstanding resistance to abiotic stress [27,28]. Some studies have indicated that quinoa has the ability to rebuild the composition and functionality of microbial communities in saline soils to cope with fluctuations in salt stress environments [4]. Peanut is a good source of protein and vegetable oil for humans [1]. With the increasing shortage of land resources, peanut is often grown in poor soil, such as saline soils [2]. However, seed germination, morphogenesis and production of peanut are severely affected by soil salinization and associated poor soil physicochemical properties [29]. Quinoa–peanut relay intercropping is a new promising planting pattern on saline–alkali lands. As “cool-loving” and “warm-loving” crops, respectively, quinoa and peanut possess different sowing dates and growing periods [30,31]. In the quinoa–peanut relay intercropping system, quinoa functions as a phytoremediation crop and is sown first in the early spring, while peanut is sown about 50 days later. Quinoa, as the crop that is sown first, needs to absorb enough water and nutrients from the soil in order to meet the needs of growth and development. While taking away part of the salt in the soil, it can change the salt content and other physicochemical properties of the soil.

To date, there have been many reports on the intercropping of peanuts with other crops, such as maize–peanut [32], cotton–peanut [2], sugarcane–peanut [33], and sorghum–peanut [20]. Researchers have studied the effects of different intercropping combinations on soil nutrient utilization and comprehensive productivity from different perspectives. However, knowledge about the effects of quinoa–peanut relay intercropping on soil desalinization and soil physicochemical and biological properties in the rhizospheric zone of peanut is still limited. And the relationships between the changes in soil properties caused by IP and the growth and pod yield of peanut are not clear. In this work, the growth and pod yield of peanut in a quinoa–peanut relay intercropping system in saline soil was evaluated. The changes in salt accumulation, the physicochemical properties of peanut rhizospheric soils at different growth stages, the correlation with the pod yield of peanut, and the microbial diversity and community composition of peanut rhizospheric soils, as well as the correlation between the soil microbial community and soil physicochemical properties, were investigated in order to provide a theoretical basis for the promotion and application of a quinoa–peanut intercropping pattern in saline–alkali soil.

## 2. Results

### 2.1. Quinoa–Peanut Relay Intercropping Alters Peanut Agronomic Characteristics and Improves Peanut Pod Yield

To assess the effects of relay intercropping on the agricultural production of peanut, the pod yield of peanut plants grown in both MP and IP was compared. We found that intercropping significantly increased the pod yield of peanut, with an 8.1% increase in 2021 and 8.5% in 2022. A significant increase in pod number, pod weight, plant biomass, and 100-kernel weight was also observed in peanut plants grown in IP. Although no difference in harvest index was found between IP and MP, the pod number, pod weight, plant biomass, harvest index, and 100-kernel weight of the peanut plants in IP increased by 7.4%, 12.2%, 11.0%, 1.2%, and 6.8% in the year 2021 and 9.3%, 12.6%, 11.9%, 0.6%, and 8.9% in the year 2022, respectively (Table 1).

**Figure 1:**
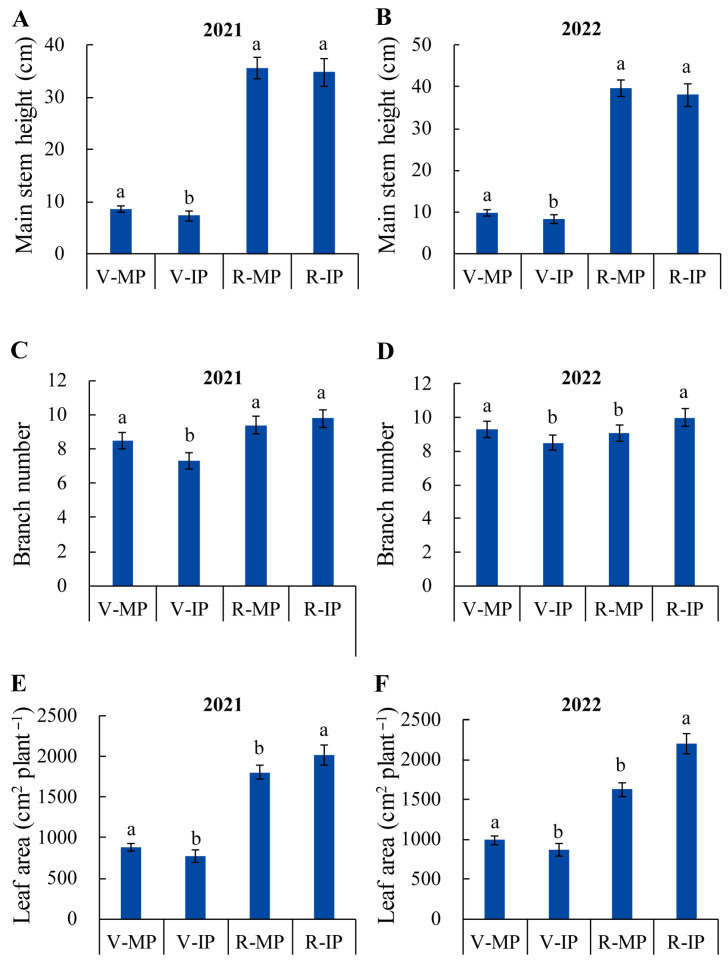
Quinoa–peanut relay intercropping altered the main stem height, branch number, and leaf area of peanut plants at both vegetative and reproductive stages. (**A**,**B**) Main stem heights of peanut plants in 2021 and 2022. (**C**,**D**) Plant branch numbers of peanut plants in 2021 and 2022. (**E**,**F**) Leaf areas of peanut plants in 2021 and 2022. MP, monocrop pattern; IP, intercrop pattern; V_MP, peanut plants in MP at vegetative stage; V_IP, peanut plants in IP at vegetative stage; R_MP, peanut plants in MP at reproductive stage; R_IP, peanut plants in IP at reproductive stage. Different lowercase letters indicate significant differences between peanut plants grown in IP and MP systems at *p* < 0.05.

Both vegetative and reproductive growth stages are crucial in the life cycle of peanut [34]. We further investigated the effects of intercropping on the agronomic traits of peanut plants at the vegetative stage (co-growth period) and reproductive stage (solo-growth period). At the vegetative stage, significant differences in main stem height, branch number, and leaf area between peanut plants grown in IP and MP systems were observed. The main stem height, branch number, and leaf area of peanut plants in IP were all lower than those of peanut plants in MP in both years of 2021 and 2022, with a 14.1% and 16.3% decrease in main stem height, 14.1% and 8.9% decrease in branch number, and 13.1% and 12.4% decrease in leaf area, respectively (Figure 1A–F). However, at the reproductive stage, the main stem height, branch number, and leaf area of peanut plants in IP all increased rapidly; the branch number and leaf area in IP were all higher than those in MP in both 2021 and 2022. It is worth noting that the leaf area of peanut plants in IP was significantly increased compared to that in MP, with an increase of 11.6% and 35.4% in 2021 and 2022, respectively (Figure 1E,F).

### 2.2. Quinoa–Peanut Relay Intercropping Changes Soil Properties

We also examined the soil physicochemical properties in the rhizosphere of peanut plants grown in both MP and IP systems. Although no difference in pH was seen, significant differences in soil properties between MP and IP were observed at both the vegetative and reproductive stages. At the vegetative stage, there was a consistent decrease in WC, EC, Na^+^, NO_3_^−^, and PO_4_^3−^ content in IP compared with those in MP in both years of 2021 and 2022. Among these parameters, the decrease in EC and Na^+^ content was most significant, with a 34.0% and 27.0% decrease in 2021 and 38.6% and 25.0% decrease in 2022, respectively (Table 2). However, the contents of K^+^, NH_4_^+^, and TOC in IP were all higher than those in MP in both 2021 and 2022, and there was a significant increase in the contents of K^+^ and TOC in IP. At the reproductive stage, soil WC and EC and the contents of K^+^, NO_3_^−^, NH_4_^+^, and PO_4_^3−^ were all clearly lower than those at the vegetative stage. Soil EC and Na^+^ content were significantly lower in IP than those in MP in both 2021 and 2022. However, soil nutrients were significantly higher in IP than those in MP, with increases of 61.0%, 10.7%, 20.4%, 12.5%, and 26.1% (2021) and 79.0%, 6.2%, 15.0%, 15.1%, and 51.6% (2022) in the contents of K^+^, NO_3_^−^, NH_4_^+^, PO_4_^3−^, and TOC, respectively.

### 2.3. A Close Relationship Among Peanut Pod Yield, Pod Dry Weight, Biomass, and Soil Properties Was Observed

The correlation coefficients among peanut pod yield, pod dry weight, plant biomass and soil property at both the vegetative and reproductive stages were analyzed. Peanut pod yield, pod dry weight, and plant biomass were all negatively correlated with WC, EC, Na^+^, NO_3_^−^, and PO_4_^3−^, and positively correlated with soil NH_4_^+^, K^+^, and TOC at the vegetative stage, with a significant correlation of pod yield and biomass with WC, and of pod yield, pod dry weight, and plant biomass with EC, Na^+^, and PO_4_^3−^ (Table 3). In addition, a significant positive correlation among pod yield, pod dry weight, and plant biomass was also observed (Table 3). Peanut pod yield, pod dry weight, and plant biomass were all negatively correlated with EC, pH, and Na^+^, and positively correlated with WC, K^+^, NO_3_^−^, NH_4_^+^, PO_4_^3−^, and TOC at the reproductive stage, with a significant correlation of pod yield, pod dry weight, and plant biomass with K^+^ and TOC, of pod yield and pod dry weight with NH_4_^+^, and of pod dry weight with WC and NO_3_^−^ (Table 4). Similarly, a significant positive correlation among pod yield, pod dry weight, and plant biomass was also observed (Table 4).

### 2.4. Quinoa–Peanut Relay Intercropping Alters Soil Bacterial Diversity in the Rhizosphere of Peanut Plants

**Figure 2:**
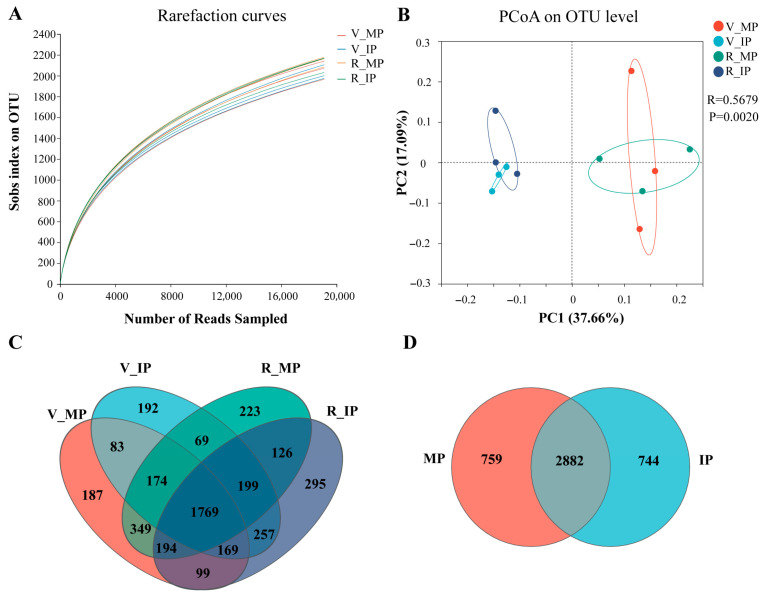
Soil bacterial diversity analysis in the rhizosphere of peanut plants in both planting patterns. (**A**) Rarefaction curve analysis showing the depth of 16S rRNA gene sequencing of the peanut rhizosphere and the possibility of observing microbial community diversity. (**B**) Principal coordinate analysis (PCoA) based on the Bray–Curtis distance demonstrating the separation between soil bacterial communities. (**C**,**D**) Venn diagrams showing the numbers of common and unique bacterial operational taxonomic units (OTUs) in the two planting patterns. MP, monocrop pattern; IP, intercrop pattern; V_MP, soil samples collected from the rhizosphere of peanut plants in MP at the vegetative stage; V_IP, soil samples collected from the rhizosphere of peanut plants in IP at the vegetative stage; R_MP, soil samples collected from the rhizosphere of peanut plants in MP at the reproductive stage; R_IP, soil samples collected from the rhizosphere of peanut plants in IP at the reproductive stage.

To see the effects of MP and IP on the community richness and diversity of the microbial ecosystem, microbial diversity was examined via alpha diversity analysis. Rarefaction curve analysis showed a high depth of 16S rRNA gene sequencing and a great possibility of observing community diversity in each peanut rhizosphere, although no differences in the sobs, Shannon, Chao 1, Ace, and Coverage indices were observed at the vegetative or the reproductive stage (Figure 2A; Table S1). To analyze the difference in bacterial communities in the two planting patterns of peanut plants at both growth stages, principal coordinate analysis (PCoA) based on the Bray–Curtis distance was performed. A clear separation was observed, with the first axis explaining 37.7% of the total variation in the bacterial community between MP and IP (Figure 2B). On the PC1 axis, samples collected from IP at both growth stages clustered closely or overlapped, while distinctly separated from those collected from MP. Combined with Anosim analysis (Bray–Curtis statistical algorithm), the β diversity of soil bacterial communities in the samples collected from IP and MP was significantly different according to bacterial community structure (*p* = 0.002, R = 0.5679). Further Venn diagram analysis demonstrated that the variation in bacterial communities in the soil samples collected from the rhizosphere of peanut plants in both IP and MP might be due to the change in the composition of a great number of shared as well as unique operational taxonomic units (OTUs). In detail, a total of 1769 common OTUs were observed in soil samples collected from the rhizosphere of peanut plants in IP and MP at both the vegetative and reproductive stages, with 192 and 187 unique OTUs in the soil samples collected from the rhizosphere of peanut plants in IP and MP at the vegetative stage, respectively. Meanwhile, at the reproductive stage, the number of unique OTUs in the soil samples in IP and MP was 295 and 223, respectively (Figure 2C). From the perspective of the overall growth period, there were 2882 common OTUs in the soil samples from IP and MP, while the unique OTUs in the soil samples from IP and MP numbered 744 and 759, respectively (Figure 2D).

### 2.5. Quinoa–Peanut Relay Intercropping Alters Soil Bacterial Community Composition in the Rhizosphere of Peanut Plants

**Figure 3:**
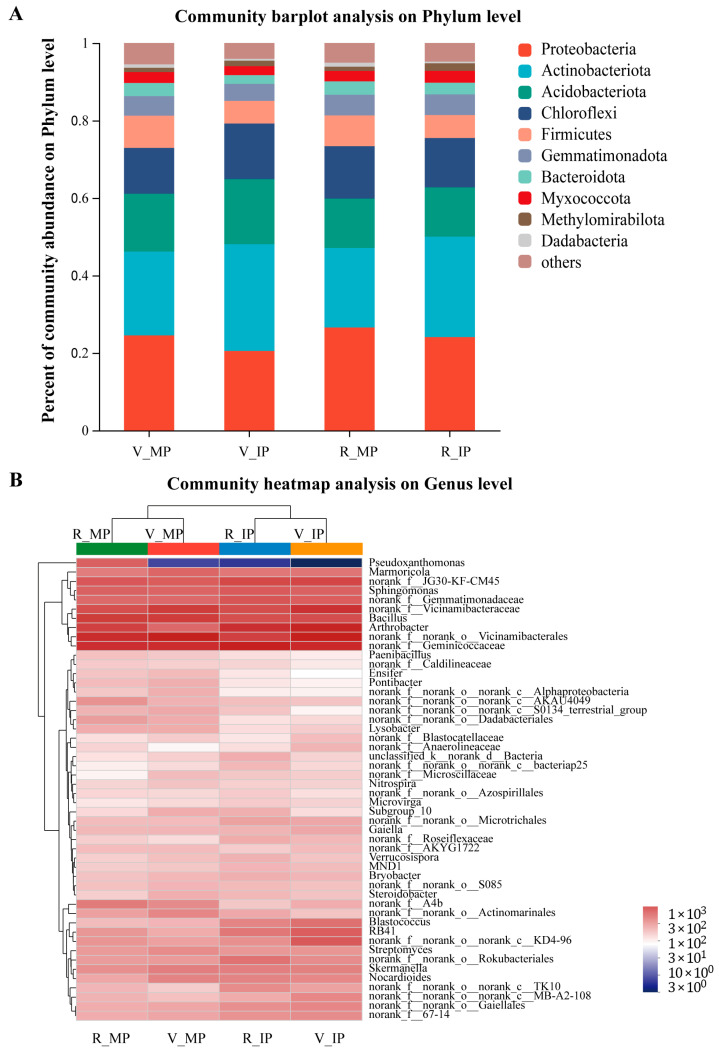
Soil bacterial community composition analysis. (**A**) Percentages of community abundance of the most dominant bacteria at the phylum level. Each color represents a phylum, and the length of the colored block represents the relative abundance ratio of species. (**B**) Taxonomic analysis at the genus level. In the phylogenetic tree and heat map, the red and blue colors, respectively, represent the more and less abundant genera in the corresponding soil samples. MP, monocrop pattern; IP, intercrop pattern; V_MP, soil samples collected from the rhizosphere of peanut plants in MP at the vegetative stage; V_IP, soil samples collected from the rhizosphere of peanut plants in IP at the vegetative stage; R_MP, soil samples collected from the rhizosphere of peanut plants in MP at the reproductive stage; R_IP, soil samples collected from the rhizosphere of peanut plants in IP at the reproductive stage.

To see the specific difference in the rhizosphere microbial communities between the two cropping systems at different growth stages of the peanut plants, we analyzed the bacterial community composition at both phylum and genus levels (Figure 3A,B). At the phylum level, the ten dominant phyla in the rhizosphere of peanut plants grown in MP and IP at both growth stages were Proteobacteria, Actinobacteriota, Acidobacteriota, Chloroflexi, Firmicutes, Gemmatimonadota Bacteroidota, Myxococcota, Methylomirabilota, and Dadabacteria, accounting for over 80% of all microbial taxa (Figure 3A). At the vegetative stage, the relative abundance of Actinobacteriota, Acidobacteriota, Chloroflexi, and Methylomirabilota in soil samples collected from the rhizosphere of peanut plants in IP was higher than that in MP. At the reproductive stage, the relative abundance of Actinobacteriota, Myxococcota, and Methylomirabilota was higher in soil samples collected from the rhizosphere of peanut plants in IP than that in MP, but the relative abundance of Proteobacteria, Firmicutes, and Bacteroidota was lower (Table S2). Further hierarchical cluster analysis at the genus level demonstrated that, based on the top 30 most abundant bacterial genera, the soil samples collected from the rhizosphere of peanut plants in MP and IP at both growth stages were clearly separated into two groups (Figure 3B). At both growth stages, the dominant genera of *norank_f__Geminicoccaceae*, *Arthrobacter*, *norank_f__Vicinamibacteraceae*, *norank_f__JG30-KF-CM45*, *Gemmatimonadaceae,* and *RB41* in IP were more abundant than those in MP (Table S3).

### 2.6. Correlation Analysis of Soil Bacterial Community Composition and Soil Properties of Peanut

**Figure 4:**
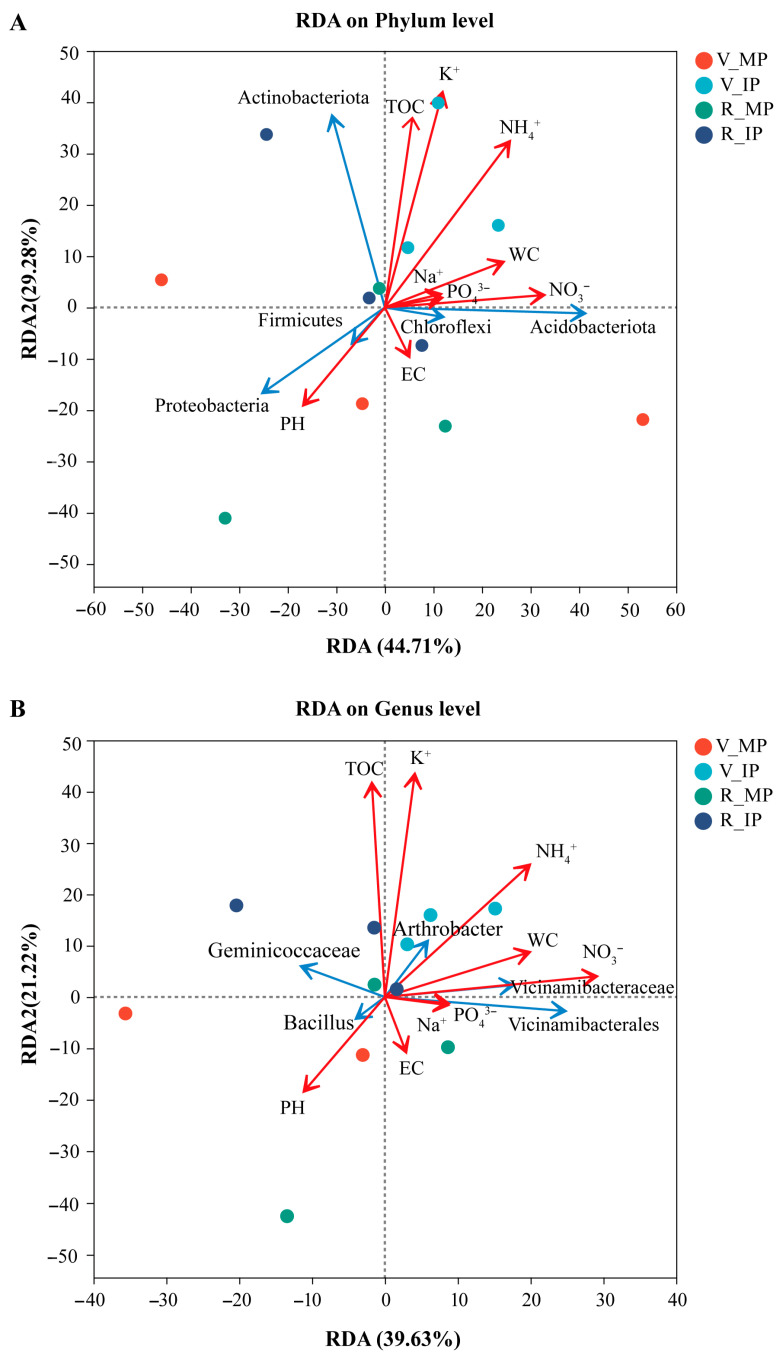
Redundancy analysis (RDA) depicting the relationship between soil properties and bacterial community. (**A**) RDA results at the phylum level. (**B**) RDA results at the genus level. MP, monocrop pattern; IP, intercrop pattern; V_MP, soil samples collected from the rhizosphere of peanut plants in MP at the vegetative stage; V_IP, soil samples collected from the rhizosphere of peanut plants in IP at the vegetative stage; R_MP, soil samples collected from the rhizosphere of peanut plants in MP at the reproductive stage; R_IP, soil samples collected from the rhizosphere of peanut plants in IP at the reproductive stage; WC, water content; EC, electrical conductivity; TOC, total organic carbon.

To understand the relationship between soil properties and bacterial communities among the soil samples collected from the rhizosphere of peanut plants in MP and IP at both growth stages, we performed RDA analysis at the phylum and genus levels. A close relationship between soil properties and bacterial communities was observed. As shown in the RDA results, percentages of 44.7% and 29.3% variation in bacterial community at the phylum level and 39.6% and 21.2% variation at the genus level are explained by the first two axes, respectively (Figure 4A,B). These results indicated that changes in soil properties played a key role in the bacterial community structure at both phylum and genus levels. In detail, at the phylum level, the relative abundance of Actinobacteriota was positively correlated with the contents of TOC, K^+^, and NH_4_^+^. The abundance of Acidobacteriota and Chloroflexi was positively correlated with the NO_3_^−^, PO_4_^3−^, Na^+^, and WC contents. And the abundance of Proteobacteria and Firmicutes was positively correlated with soil pH values (Figure 4A). At the genus level, the relative abundance of *Vicinamibacterales* and *Vicinamibacteraceae* was positively correlated with PO_4_^3−^, NO_3_^−^, and WC contents. The abundance of *Arthrobacter* was positively correlated with the K^+^, NH_4_^+^, and TOC contents. And the relative abundance of *Bacillus* was positively correlated with soil pH values (Figure 4B).

## 3. Discussion

### 3.1. Growth Dynamics and Yield Performance of Peanut

Peanut is one of the most commonly used intercropping crops in different combinations, such as maize–peanut, sorghum–peanut, sugarcane–peanut, and cotton–peanut [17,20,33,35]. In these intercropping combinations, the two crops are simultaneously cultivated. Compared to other companion crops, peanut is a shorter crop which usually experiences a negative effect due to the shading from the border rows of its companion crops, leading to a lack of advantage for its productivity. In this study, we established a new quinoa–peanut relay intercropping system in which the early maturing variety of quinoa was first sowed in strips in early spring and about 50 days later, and peanuts were sowed in strips within the reserved space in saline–alkali soils. The overlapping period of quinoa and peanut lasted about 50 days. Based on our two-year field experiments, the relay intercropping system significantly increased the pod yield of peanut per unit area. The improved pod yield of peanut plants mainly resulted from the increased pod weight, biomass, pod number per plant, and seed weight (Table 1). The growth and development advantages of peanut plants between MP and IP varied at different growth stages. At the vegetative stage, IP showed a certain disadvantage, while at reproductive stage, IP showed obvious advantages in terms of branch number and leaf area compared with MP, and there was more peanut pod productivity in IP (Figure 1; Table 1). These results are different from the observations reported in previous studies. Most studies have shown that the pod yield of peanut in an intercropping system is not increased, or even decreases to some extent, compared with that in a monoculture system, and in these intercropping combinations, the growth of peanut is mostly inhibited during the growth period [33,35,36]. In the symbiotic intercropping of maize and ruzi grass, the growth and yield of crops are dependent on the symbiotic complementarity of plant species, planting pattern, and overlapping period [37]. In the symbiotic intercropping of proso millet and mung bean, the combined crops compete with each other for light and nutrient resources, and sometimes, their competitive ability shifts with the aboveground and underground ecological niches. In our quinoa–peanut relay intercropping system, the quinoa was grown as a tall and early sowing crop, and it exhibited an evident growth advantage due to the fact that the quinoa plants were well developed (heading stage) [38] when the peanuts were sowed. As a late-sown crop, peanut displays weak competition at the early stage of growth and the growth of it is usually competitively inhibited by quinoa. However, we selected short-stemmed and early-maturing quinoa varieties in order to reduce the intensity and duration of competition between the quinoa and peanuts. On the contrary, for peanut, mid- and late-maturing varieties were selected to extend the recovery growth period of peanut. In this way, the maximum nutrient demand periods of quinoa and peanut have a greater complementary advantage in temporal ecological niches in the system. They have separate peak nutrient demand periods. Therefore, the competitive effects of quinoa on peanut are temporal. After the quinoa matured and was harvested, the peanut entered its flowering and needle stage, which is the most critical period for the increase in pod yield [39]. Peanut plants in IP exhibited fast recovery growth, as indicated by the rapid increase in branch number and leaf area, which compensated for the disadvantage in the early growth period. In particular, the significant increase in leaf area in IP at the late growth stage obviously delays the premature senescence of peanut, which is conducive to the accumulation of photosynthetic products and improvement of pod yield [40]. The competition recovery of peanut plants in IP at the reproductive stage could be related to the higher aboveground radiation use efficiency after the quinoa was harvested, and the more efficient utilization of nutrient resources underneath quinoa plants.

### 3.2. Soil Physicochemical Properties and Their Relationship with the Pod Yield of Peanut

In most cases, soil salinization occurs as severe form of environmental stress [41]. During the production of peanut in saline–alkali soil, excessive salt ions and associated extreme conditions in the soil severely affect peanut productivity, especially at the seedling stage [41]. In this study, we used quinoa–peanut relay intercropping to reduce soil salinity and improve soil physicochemical properties. Quinoa is a crop with strong salt tolerance, and is able to absorb a great amount of salt ions during the overlapping period in an intercropping system [42]. We found that quinoa–peanut relay intercropping significantly decreased soil WC, EC, and Na^+^ contents in the rhizosphere of peanut plants at both the vegetative and reproductive stages (Table 2). The results of correlation analysis show that there was a significant positive correlation between soil EC and soil WC. The reason may be that intercropped quinoa absorbed soil water while taking away some salt from the soil, therefore reducing the soil salt content. Moreover, the biomass, pod dry weight, and pod yield of peanut plants were all significantly and negatively correlated with soil WC, EC, and Na^+^ contents in the rhizosphere of peanut plants at the vegetative stage (Table 3). These results indicated that the decreased soil EC and Na^+^ contents in IP at the early growth stage of the peanut were helpful to the increase in its pod yield.

Soil nutrients are an important factor limiting the productivity of intercropping crops, and N, P, and K are three essential macronutrients for crop growth and yield production [43,44]. Some studies show that intercropping increases the soil available nutrients of peanut compared with monoculture [2,33], while some studies have also shown that intercropping reduces soil available nutrients [45]. In this study, the effects of quinoa–peanut relay intercropping on soil available nutrient content and soil fertility varied with different nutrient types and specific growth stages. At the vegetative stage of peanut, the contents of soil NO_3_^−^ and PO_4_^3−^ in the rhizosphere of the peanut plants in IP were lower than those in MP, but the contents of K^+^, NH_4_^+^, and TOC in IP were higher. At the reproductive stage of peanut, the contents of soil NO_3_^−^, PO_4_^3−^, K^+^, NH_4_^+^, and TOC in IP were all higher than those in MP (Table 2). The differences and dynamic changes in rhizosphere soil nutrients of intercropping crops are related to the interaction of root competition, root exudates, microbial community structure, and metabolites [17,33,46]. At the vegetative stage, quinoa is in the grain-filling stage, which is a period of greater nutrient demand. The well-developed root system of quinoa has more nutrient-competitive advantages compared to peanut at the seedling stage in an intercropping system. Therefore, root competition may be the dominant factor in the nutrient change of rhizosphere soil during this period. At the reproductive stage, the intensity of nutrient competition for peanuts will be reduced because the quinoa has been harvested. Under the comprehensive influence of root exudates, microorganisms, and other environmental factors, the soil available nutrients of the peanut rhizosphere changed in this period. The higher NH_4_^+^, K^+^, and TOC contents of the peanut rhizosphere soil in IP were significantly positively correlated with the pod yield of peanut (Table 4). This indicated that the greater nutrient resource at the reproductive stage of peanut in IP was an important factor in increasing the peanut pod yield.

### 3.3. Soil Microbial Community Characteristics and Their Relationships with Soil Properties

The soil bacterial community plays a crucial role in maintaining the stability of an agricultural ecosystem [47]. Increasing the species diversity and improving the planting structure can improve the soil microbial community characteristics [48,49]. Some studies show that intercropping increases soil microbial diversity and changes the microbial community structure [48,50]. However, some studies also show that intercropping only limitedly changes soil bacterial community diversity and richness [2,47]. Similarly, in our study, quinoa–peanut relay intercropping did not increase the bacterial sobs index or Shannon index in the rhizosphere of peanut plants, as indicated by the results of the alpha diversity analysis (Table S1). The difference may be due to the changes in soil microenvironment caused by interspecific interactions, which determined the differential selection of microbial partners from the soil environment [20]. Distinct differences in bacterial community structure between IP and MP were observed in our study (Figure 2A). A remarkable increase in the relative abundance of some beneficial dominant and functional bacterial communities at the phylum level, such as Actinobacteriota, Acidobacteriota, Chloroflexi, and Methylomirabilota, was observed in the rhizosphere of peanut plants in IP at the vegetative stage (Figure 3A; Table S2). Most of these microorganisms were beneficial to soil fertility [51,52]. Actinobacteriota and Methylomirabilota are important for C cycling, and the increase in the relative abundance of them could have significant positive effects on the formation of organic carbon [53,54]. It can be seen from the RDA results that the abundance of Actinobacteriota and Methylomirabilota was positively correlated with TOC, indicating that the increase in the relative abundance of Actinobacteriota and Methylomirabilota was helpful to the increase in soil TOC (Figure 4A). Acidobacteria have many beneficial activities, such as the use of nitrite as a N source, the response to soil macro- and micronutrients and soil acidity, the expression of multiple active transporters, the degradation of gellan gum, and the production of exopolysaccharide [52,55]. Consistently, we found that higher NO_3_^−^ content in the peanut rhizosphere soil was positively correlated with an abundance of Acidobacteria (Figure 4A).

In terms of bacterial community structure, a clear distinction between IP and MP was observed at the genus level. Most of the dominant genera, such as *norank_f__Geminicoccaceae*, *Arthrobacter*, *norank_f__Vicinamibacteraceae*, *JG30-KF-CM45*, *norank_f__Gemmatimonadaceae*, and *RB41,* in IP were more abundant than in MP (Figure 3B). The community composition of rhizosphere soil microorganisms is dependent on the plant species, soil type, plant developmental stage, and agricultural management [56]. Among them, soil salinity is a major factor that drives plant–soil feedback by modifying microbial communities [57]. In the quinoa–peanut intercropping system, the salt content and physicochemical properties of peanut rhizospheric soil changed significantly compared with those of monoculture peanut due to the presence of quinoa (Table 2). Based on the specific environments, rhizosphere microorganisms co-evolve with their host to adjust to the community structure. Previous studies show that *Vicinamibacteraceae* encodes a pit that participates in the assimilation of phosphate and prevents the loss of available phosphorus in an extreme leaching environment, and the *Arthrobacter* species plays important roles in the catabolism of environmental pollutants [58,59]. Our RDA analysis further confirmed this conclusion; the abundance of *norank_f__Vicinamibacteraceae* was positively correlated with the content of nutrients such as NO_3_^−^ and PO_4_^3−^, and the abundance of *Arthrobacter* was positively correlated with the nutrient contents of K^+^, NH_4_^+^, and TOC (Figure 4B). These results indicate that the increase in the relative abundance of the mentioned microbial communities in IP was beneficial to soil nutrient activation and metabolism. However, the rebuilding of rhizosphere microbial community structure is the result of a series of complex interactions and feedback loops among plant roots, microorganisms, and soil physicochemical factors [60]. The interaction mechanism between root systems, soil physicochemical properties, and microbial community composition in quinoa–peanut relay intercropping systems remains to be further elucidated.

## 4. Materials and Methods

### 4.1. Field Experimental Site

**Figure 5:**
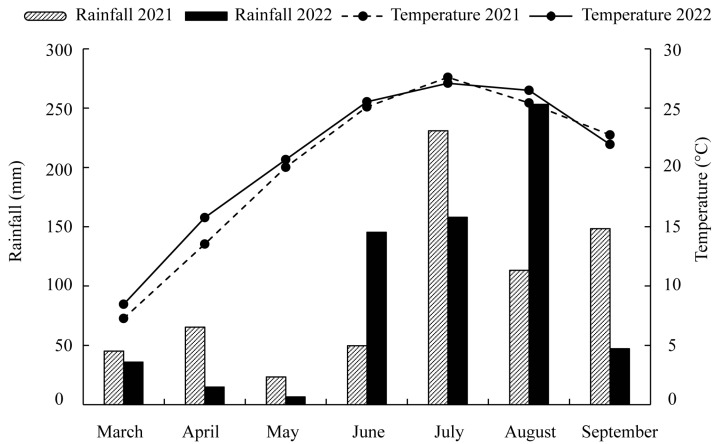
Monthly rainfall and average temperature during the intercropping seasons in 2021 and 2022.

Field experiments were conducted in 2021 and 2022 at the Yellow River Delta Modern Agriculture Experimental and Demonstration Base of Shandong Academy of Agricultural Sciences (118.37 °E, 37.18 °N), Shandong Province, China. The field test sites were located in a warm semi-arid monsoon region with a continental climate during spring and autumn. The monthly average temperature and precipitation for the years of 2021 and 2022 in the intercropping seasons (March–September) are shown in Figure 5. Total rainfall and monthly average temperatures between 2021 and 2022 were similar. Rainfall in 2021 was mainly focused in July, August, and September, while rainfall in 2022 was mainly focused in June, July, and August. Sufficient precipitation at the early stage in 2022 was beneficial to the growth of the peanut plants. The slightly higher mean temperature in May and June in 2022 was beneficial to the seedling growth of peanut. The top soil’s (0–20 cm layer) properties before the experiments were as follows: pH of 7.96, EC of 768.4 μs/cm, total nitrogen of 1.03 g/kg, total phosphate of 739.01 mg/kg, total potassium of 19.17 g/kg, available nitrogen of 50.22 mg/kg, available phosphorus of 27.12 mg/kg, available potassium of 119.57 mg/kg, and organic carbon of 13.91 g/kg.

### 4.2. Experimental Design and Field Management

Two peanut planting patterns, the traditional monocrop pattern (MP) and a quinoa–peanut relay intercrop pattern (IP), were arranged. The two planting patterns were designed using a randomized complete block with three replications consisting of 6 plots. The area of each plot was 7 × 25 m (175 m^2^), and a protection zone with a width of 1 m was arranged between adjacent plots to eliminate the impact of the lateral movement of soil water and salinity. Seeds of the commercial peanut cultivar Huayu 25 were generously provided by the Shandong Peanut Research Institute, Shandong Academy of Agricultural Sciences (Qingdao, China), and seeds of the quinoa cultivar Huaqing 1 were purchased from Shanxi Huaqing Quinoa Product Development Co., Ltd. (Xinzhou, China). The quinoa and peanut varieties have a growth period of 100 days and 120 days, respectively.

**Figure 6:**
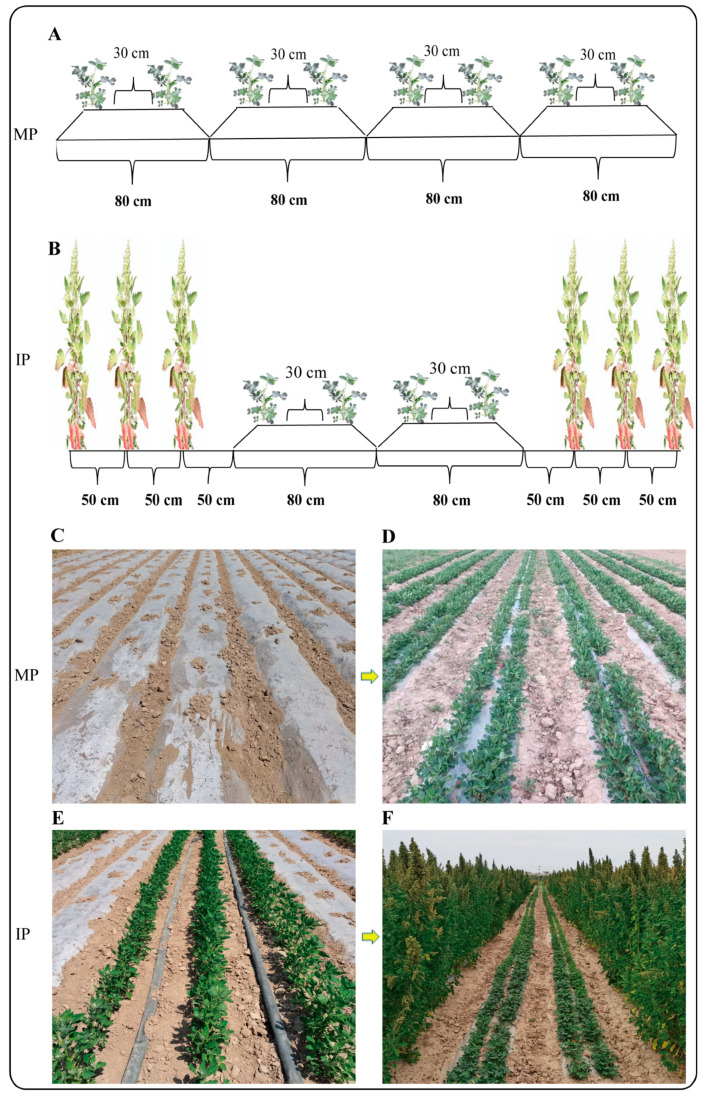
Schematic diagrams and field pictures showing the monocropping of peanut and the relay intercropping of quinoa and peanut. (**A**) Schematic diagram showing the traditional monocropping pattern of peanut. (**B**) Schematic diagram showing the relay intercropping of quinoa and peanut. (**C**) Image showing the monocropping of peanut at the sowing stage in the field. (**D**) Image showing the monocropping of peanut at the seedling stage in the field. (**E**) Image showing the relay intercropping of quinoa and peanut at the sowing stage of peanut and seedling stage of quinoa in the field. (**F**) Image showing the relay intercropping of quinoa and peanut in the overlapping period in the field. MP, monocrop pattern; IP, intercrop pattern.

For the intercropping, three rows of quinoa were sowed first in March (18 March in 2021 and 15 March in 2022) with a row space of 50 cm and plant distance of 25 cm, and peanut was sowed in May (6 May in 2021 and 8 May in 2022) with two rows on each ridge, with a ridge width of 50 cm and furrow width of 30 cm. The row space on the same ridge was 30 cm and the plant distance was 15 cm. The space between the quinoa and peanut was 65 cm. Each intercropping zone consisted of four rows of peanut and three rows of quinoa. The quinoa was harvested on 28 June in 2021 and 26 June in 2022, and peanuts were harvested on 8 September in 2021 and 12 September in 2022. The overlapping period of quinoa and peanut was about 50 days. For the traditional monocrop planting of peanut, the sowing time and method were the same as those for the relay intercropping. Peanut plants in the two patterns were covered with film on the ridge. Schematic diagrams and field pictures showing the two planting patterns are shown in Figure 6. In each experimental plot, rotten chicken manure (225 kg·ha^−1^) was applied in the winter of the year before sowing. In addition, 600 kg·hm^−2^ of compound fertilizer (N-P_2_O_5_-K_2_O: 15-15-15) was applied in the quinoa planting belt as a basal fertilizer before the sowing of the quinoa, and 90 kg·hm^−2^ of nitrogen (N), 120 kg·hm^−2^ of phosphorus (P_2_O_5_), 150 kg·hm^−2^ of potassium (K_2_O), and 90 kg·hm^−2^ of slow-release nitrogen fertilizer were applied in the peanut planting belt as a basal fertilizer before the sowing of the peanut. The total amount of irrigation water in each cropping pattern was the same during the growing season of peanut, with irrigation in each treatment. All the other cultivation and management measures were the same during the growing stage of the peanut plants.

### 4.3. Plant Sampling and Pod Yield

For the determination of main stem height, branch number, and leaf area per plant, twelve uniform peanut plants from the monocrop pattern and twelve from the two borders and two inner rows (three plants from each row) in the relay intercropping pattern were, respectively, sampled at both the vegetative (40 days after sowing) and reproductive (85 days after sowing) stages. The leaf area was determined as described previously [61]. Essentially, one leaf disc with a diameter of 1.0 cm was punched for every third leaf from the top of the peanut plants, and thirty leaves were sampled. The leaf discs and samples were dried at 75 °C to a constant weight. Then, the leaf dry weight was determined and leaf area was calculated as follows: LA = LA_disc_/DM_disc_ × DM_total_ (cm^2^). For pod yield assays, pods from peanut plants from 4 square meters in the two borders and two inner rows in the intercropping system, as well as pods from the monocropping system, were manually harvested; simultaneously, the pod number per plant was recorded. After the pods were sun-dried for 15 days, the pod weight per plant, biomass per plant, and pod yield were recorded. The harvest index is equal to the ratio of pod weight to biomass of peanut. At the same time, 100-kernel weight was determined.

### 4.4. Soil Sampling

To collect soil samples from the rhizosphere corresponding to the plant samples in different plots at both the vegetative and reproductive stages, plants with rhizosphere soil attached were dug out with a spade sanitized with 70% alcohol. Before collecting the next soil sample, the spade was sanitized with 70% alcohol and wiped with sterile paper to avoid contamination from the previous sample. Rhizosphere soil tightly attached to plant roots was gently shaken off the plants and sieved through a 2 mm mesh to remove stones and other residues. Rhizosphere soils collected from every four plants from different rows were pooled together as one replicate. Three replicates were performed for each treatment. For the analysis of soil properties, the soil samples were air-dried. For molecular analyses, the soil samples were immediately homogenized in liquid nitrogen and stored at −80 °C.

### 4.5. Soil Physicochemical Property Analyses

For soil water content (WC) assays, the soil samples were dried in aluminum boxes at 105 °C for 10 h. For physicochemical property analyses, the soil samples were air-dried first, then ground and sieved through 0.25 mm, 1 mm, and 2 mm meshes. The soil sample sieved with a 2.0 mm mesh was used to determine the soil pH and electrical conductivity (EC). The soil pH and EC were measured using a pH meter (Seven Compact S210, Mettler Toledo, Shanghai, China) and a digital conductivity meter (DDS-11A, Shanghai Precision & Scientific Instrument Co., Ltd., Shanghai, China) at a soil/water ratio of 1:5 after shaking the mixture for approximately 30 min, as described previously [62]. The soil sample sieved with a 1 mm mesh was used to determine nitrate-nitrogen (NO_3_^−^-N), ammonium nitrogen (NH_4_^+^-N), and phosphate ion (PO_4_^3−^). Essentially, NO_3_^−^-N, NH_4_^+^-N, and PO_4_^3−^ were extracted with 2 mol L^−1^ KCl and measured with a continuous flow analyzer (AutoAnalyzer-AA3, Seal Analytical, Norderstedt, Germany). The soil samples passed through the 0.25 mm mesh were used to determine the soil total organic carbon (TOC) using the Walkley–Black method, as described previously [63]. Soil ion (K^+^, Na^+^) contents were analyzed using a flame atomic absorption spectrophotometer Cole-Parmer FF-200D (Cole-Parmer, Cambridge, UK), as described by Sun et al. [64].

### 4.6. Soil DNA Extraction, PCR Amplification, and Sequencing Analysis

The genomic DNA of the microbial community was isolated from the rhizospheric soils of peanut in 2022 with the E.Z.N.A.^®^ soil DNA Kit (Omega Bio-tek, Norcross, GA, USA). The quality of DNA was assessed on 1.0% agarose gel, and the concentration and purity of the DNA were then checked with a NanoDrop 2000 UV-vis spectrophotometer (Thermo Scientific, Wilmington, DE, USA). The V3-V4 hypervariable region of the bacterial 16S rRNA gene was amplified using primers 338F (5′-ACTCCTACGGGAGGCAGCAG-3′) and 806R (5′-GGACTACHVGGGTWTCT AAT-3′) with the ABI GeneAmp^®^ 9700 PCR thermocycler (Applied Biosystems, Foster City, CA, USA). PCR amplification of the 16S rRNA gene was conducted as follows: initial denaturation at 95 °C for 3 min, followed by 27 cycles of denaturation at 95 °C for 30 s, annealing at 55 °C for 30 s and extension at 72 °C for 45 s, single extension at 72 °C for 10 min, and ending at 4 °C. The PCR reaction buffer contained 4 μL of 5× TransStart FastPfu buffer, 2 μL of 2.5 mM dNTPs, 0.8 μL of forward primer (5 μM), 0.8 μL of reverse primer (5 μM), 0.4 μL of TransStart FastPfu DNA Polymerase, and 10 ng of template DNA, and we used dd H_2_O to adjust to 20 μL.

### 4.7. Llumina MiSeq and Processing of Sequencing Data

The resultant PCR products isolated from the 2% agarose gel were purified with the AxyPrep DNA Gel Extraction Kit (Axygen Biosciences, Union City, CA, USA) following the manufacturer’s instructions. Purified amplicons were combined into equimolar and paired-end sequences on an Illumina MiSeq PE300 platform/NovaSeq PE250 platform (Illumina, San Diego, CA, USA) following the standard protocol (Majorbio Bio-Pharm Technology Co., Ltd. Shanghai, China). Raw reads were deposited into the NCBI Sequence Read Archive (SRA) database with the accession number PRJNA1091820. Raw 16S rRNA gene sequencing reads were demultiplexed, quality-filtered, and merged using FLASH v.1.2.7. Operational taxonomic units (OTUs) with a 97% similarity cut-off were clustered using UPARSE v.7.1, and chimeric sequences were identified and removed [65]. The taxonomy of each OTU representative sequence was analyzed with RDP Classifier v.2.2 against the 16S rRNA database using a confidence threshold of 0.7. Soil microbial communities were identified and annotated at the phylum and genus levels as described previously [66].

### 4.8. Statistical Analysis

SPSS software version 19.0 (SPSS, Inc., Chicago, IL, USA) was used to perform the statistical analyses. One-way analysis of variance (ANOVA) and the least significant difference (LSD) multiple-range test (*p* < 0.05) were used to statistically analyze the differences across the treatments. Microbial community analyses were conducted on the Majorbio Cloud Platform. The Mothur software (v.1.30.2) was used to calculate the α-diversity. Rarefaction curves were generated using Mothur at a 97% identity level. Venn and bar diagrams were generated with R script (v.3.3.1). Beta diversities were visualized via principal coordinate analysis (PCoA) based on the Bray–Curtis distance. Redundancy analysis (RDA) was conducted with the software R (version 3.3.1) and graphed using the vegan package.

## 5. Conclusions

**Figure 7:**
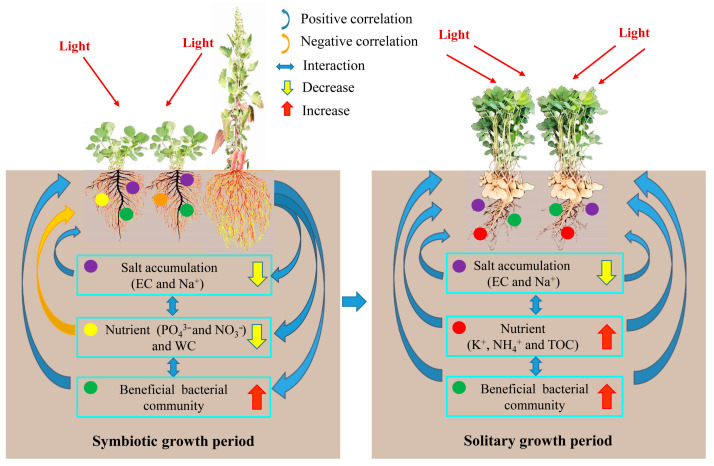
Mechanism of improvement of peanut productivity in quinoa–peanut relay intercropping system.

Quinoa–peanut relay intercropping promoted peanut productivity in saline–alkali soil. Although there was a disadvantage in terms of plant development at the early growth stage, the pod yield of the peanut plants per unit area in IP was remarkably improved compared with that of the peanut plants in MP. The increase in pod yield in IP was significantly and positively correlated with the decrease in salt accumulation, such as soil EC and Na^+^ contents, and the increase in soil nutrients, such as soil K^+^, NH_4_^+^, and TOC contents, in the rhizosphere of peanut plants at the reproductive stage. In addition, quinoa–peanut relay intercropping recruited more beneficial functional bacteria in the rhizosphere of peanut plants, which might be helpful for the improvement of soil nutrient availability. Overall, quinoa–peanut relay intercropping improved peanut productivity by decreasing soil salt accumulation and by regulating soil nutrient utilization and microbial community composition. The specific principle is shown in Figure 7. This planting pattern could be recommended as an economical system suitable for use on saline–alkali land in the North China Plain, such as in the Yellow River Delta and other regions with similar ecological conditions.

## Figures and Tables

**Table 1:** Effects of quinoa–peanut relay intercropping on peanut growth and pod yield.

Year	Treatment	Pod Yield (kg/hm^2^)	Pod Number/plant	Pod Weight (g/plant)	Biomass (g/plant)	Harvest Index	100 Kernel Weight (g)
2021	MP	6304 ± 20 b	18.8 ± 1.0 b	29.6 ± 1.6 b	58.3 ± 3.0 b	0.508 ± 0.02 a	86.5 ± 3.5 b
	IP	6814 ± 21 a	20.2 ± 1.2 a	33.2 ± 1.7 a	64.7 ± 4.2 a	0.513 ± 0.03 a	92.4 ± 4.0 a
2022	MP	7124 ± 29 b	22.5 ± 1.2 b	36.0 ± 1.8 b	69.0 ± 3.8 b	0.521 ± 0.01 a	87.7 ± 4.2 b
	IP	7730 ± 29 a	24.6 ± 1.3 a	40.48 ± 2.1 a	77.2 ± 4.3 a	0.524 ± 0.02 a	95.5 ± 3.6 a

Data are mean ± SE (n = 3). Different lowercase letters within a column represent significant differences (*p* < 0.05). MP, monocrop pattern; IP, intercrop pattern.

**Table 2:** Effects of quinoa–peanut relay intercropping on soil properties in the rhizosphere of peanut plants.

Year	Treatment	WC %	EC (us/cm)	pH	Na^+^ (mg/kg)	K^+^ (mg/kg)	NO_3_^−^ (mg/kg)	NH_4_^+^ (mg/kg)	PO_4_^3−^ (mg/kg)	TOC (g/kg)
2021	V_MP	31.4 a	682.4 a	7.93 a	282.5 a	283.7 b	20.5 a	11.9 a	82.3 a	8.64 b
	V_IP	27.7 b	450.2 b	7.98 a	206.1 b	364.3 a	18.7 b	12.1 a	70.7 b	9.43 a
	R_MP	15.1 a	386.6 a	8.06 a	132.8 a	179.6 b	13.7 b	7.3 b	35.8 b	6.70 b
	R_IP	14.9 a	304.2 b	8.04 a	118.2 b	289.2 a	15.1 a	8.7 a	40.3 a	8.45 a
2022	V_MP	22.3 a	576.7 a	7.91 a	244.1 a	274.7 b	22.4 a	12.6 b	89.6 a	8.36 b
	V_IP	19.3 b	354.2 b	7.96 a	183.1 b	341.9 a	19.6 b	14.1 a	66.1 b	9.58 a
	R_MP	12.3 a	297.2 a	8.13 a	102.4 a	157.7 b	12.8 b	8.2 b	38.0 b	6.13 b
	R_IP	13.7 a	230.9 b	8.09 a	88.1 b	282.2 a	13.6 a	9.4 a	43.7 a	8.62 a

Note: Data are mean ± SE (n = 3). Different lowercase letters within a column represent significant difference (*p* < 0.05). WC, soil water content; EC, soil electrical conductivity; TOC, total organic carbon; MP, monocrop pattern; IP, intercrop pattern. V_MP, soil samples collected from the rhizosphere of peanut plants in MP at the vegetative stage; V_IP, soil samples collected from the rhizosphere of peanut plants in IP at the vegetative stage; R_MP, soil samples collected from the rhizosphere of peanut plants in MP at the reproductive stage; R_IP, soil samples collected from the rhizosphere of peanut plants in IP at the reproductive stage; WC, water content; EC, electrical conductivity; TOC: total organic carbon.

**Table 3:** Correlation coefficients between pod yield, pod dry weight and biomass of peanut at podding stage and physicochemical properties of rhizosphere soil of peanut plants at vegetative stage.

	WC	EC	pH	Na^+^	K^+^	NO_3_^−^	NH_4_^+^	PO_4_^3−^	TOC	PY	PDW	BI
WC	1											
EC	0.859 *	1										
pH	−0.754	−0.828 *	1									
Na^+^	0.78	0.948 **	−0.772	1								
K^+^	−0.775	−0.686	0.537	−0.507	1							
NO_3_	0.834 *	0.501	−0.329	0.432	−0.539	1						
NH_4_^+^	−0.508	−0.471	0.586	−0.264	0.278	−0.434	1					
PO_4_^3−^	0.919 **	0.951 **	−0.892 *	0.832 *	−0.792	0.578	−0.609	1				
TOC	−0.757	−0.575	0.634	−0.399	0.932 **	−0.515	0.337	−0.761	1			
PY	−0.828 *	−0.978 **	0.899 *	−0.942 **	0.681	−0.401	0.413	−0.947 **	0.624	1		
PDW	−0.719	−0.922 **	0.684	−0.917 *	0.712	−0.311	0.119	−0.823 *	0.555	0.927 **	1	
BI	−0.906 *	−0.885 *	0.592	−0.856 *	0.798	−0.706	0.21	−0.841 *	0.654	0.838 *	0.885 *	1

Note: WC, soil water content; EC, soil electrical conductivity; TOC, total organic carbon; PY, pod yield of peanuts; PDW, pod dry weight per plant; BI, biomass per plant. * and ** represent significant difference at *p* < 0.05 and *p* < 0.01, respectively.

**Table 4:** Correlation coefficients between pod yield, pod dry weight and biomass of peanut at podding stage and physicochemical properties of rhizosphere soil of peanut plants at reproductive stage.

	**WC**	**EC**	**pH**	**Na^+^**	**K^+^**	**NO_3_^−^**	**NH_4_^+^**	**PO_4_^3−^**	**TOC**	**PY**	**PDW**	**BI**
WC	1											
EC	−0.205	1										
pH	−0.712	0.104	1									
Na^+^	−0.568	0.735	0.452	1								
K^+^	0.684	−0.681	−0.735	−0.638	1							
NO_3_^−^	0.913 *	−0.331	−0.663	−0.756	0.650	1						
NH_4_^+^	0.709	−0.414	−0.866 *	−0.441	0.945 **	0.601	1					
PO_4_^3−^	0.503	−0.615	−0.002	−0.347	0.572	0.476	0.404	1				
TOC	0.779	−0.691	−0.641	−0.673	0.965 **	0.755	0.878 *	0.735	1			
PY	0.752	−0.422	−0.771	−0.430	0.920 **	0.698	0.945 **	0.594	0.918 **	1		
PDW	0.891 *	−0.45	−0.637	−0.536	0.852 *	0.837 *	0.823 *	0.759	0.940 **	0.927 **	1	
BI	0.672	−0.808	−0.483	−0.731	0.910 *	0.726	0.754	0.803	0.969 **	0.838 *	0.885 *	1

Note: WC, soil water content; EC, soil electrical conductivity; TOC, total organic carbon; PY, pod yield of peanuts; PDW, pod dry weight per plant; BI, biomass per plant. * and ** represent significant difference at *p* < 0.05 and *p* < 0.01, respectively.

## Data Availability

All raw reads were deposited in the Sequence Read Archive (SRA) database in NCBI, with the accession number PRJNA1091820.

## Supplementary Materials

The following supporting information can be downloaded at: https://www.mdpi.com/article/10.3390/plants14142102/s1, Table S1: The effect of quinoa–peanut relay intercropping on the soil bacterial diversity index of peanut; Table S2: The effect of quinoa–peanut relay intercropping on the relative abundances (%) of bacterial communities at the phylum level; Table S3: The effect of quinoa–peanut relay intercropping on the relative abundances (%) of bacterial communities at the genus level.
