# Supplementary material for: Quinoa–Peanut Relay Intercropping Promotes Peanut Productivity Through the Temporal Optimization of Soil Physicochemical Properties and Microbial Community Composition in Saline Soil
Source: Plants (Basel). 2025 Jul 8;14(14):2102. doi: 10.3390/plants14142102 (PMC12298141; doi:10.3390/plants14142102)
Supplement: Supplementary file 1 [file plants-14-02102-s001.zip › plants-3685867-supplementary.pdf]

Table S1. Effect of quinoa-peanut relay intercropping on the soil bacterial diversity index of peanut.

Period	Treatment	Sobs	Shannon	Chao 1	Ace	Coverage
VP	V_MP	2059.3 ± 112a	6.42 ± 0.33a	2798.2 ± 142a	2812.2 ± 143a	0.963 ± 0.049a
	V_IP	2026.3 ± 98a	6.32 ± 0.30a	2848.5 ± 138a	2841.3 ± 140a	0.962 ± 0.048a
RP	R_MP	2141.0 ± 109a	6.42 ± 0.30a	2905.9 ± 145a	2948.7 ± 152a	0.961 ± 0.048a
	R_IP	2122.0 ± 110a	6.43 ± 0.32a	2903.7 ± 146a	2912.7 ± 136a	0.962 ± 0.049a

Values followed by different letters within a column are significantly different at $P < 0.05$.

Table S2. Effect of quinoa-peanut relay intercropping on the relative abundances (%) of bacterial community on the phylum level.

OTU ID	V_MP	V_IP	R_MP	R_IP
Proteobacteria	24.53a	20.51b	26.59a	24.09b
Actinobacteriota	21.64b	27.57a	20.52b	25.93a
Acidobacteriota	14.92b	16.77a	12.73a	12.72a
Chloroflexi	11.83b	14.38a	13.53a	12.76b
Firmicutes	8.31a	5.84b	7.95a	5.91b
Gemmatimonadota	5.07a	4.39b	5.33a	5.33a
Bacteroidota	3.39a	2.26b	3.47a	3.04b
Myxococcota	2.80a	2.33b	2.68b	3.06a
Methylomirabilota	1.13b	1.39a	1.10b	1.94a
Dadabacteria	0.87a	0.49b	1.05a	0.44b
others	5.50a	4.07b	5.06a	4.79b

Values followed by different letters within a column are significantly different at $P < 0.05$.

Table S3. Effect of quinoa-peanut relay intercropping on the relative abundances (%) of bacterial community on the genus level.

OTU ID	V_MP	V_IP	R_MP	R_IP
Vicinamibacterales	5.47a	5.54a	4.56a	3.59b
Geminicoccaceae	4.50b	4.73a	4.40b	5.32a
Arthrobacter	2.11b	5.08a	3.59b	4.35a
Vicinamibacteraceae	3.75b	4.33a	2.74b	2.97a
Bacillus	3.81a	3.03b	3.78a	2.91b
JG30-KF-CM45	2.50b	3.31a	2.59b	3.27a
Sphingomonas	2.21a	2.33a	2.35a	2.52a
Gemmatimonadaceae	2.10b	2.43a	2.05b	2.70a
Marmoricola	2.20a	1.79b	1.61a	1.72a
RB41	0.89b	2.43a	1.15b	1.72a

Values followed by different letters within a column are significantly different at $P < 0.05$.
